# Cardiorenal Involvement in Metabolic Syndrome Induced by Cola Drinking in Rats: Proinflammatory Cytokines and Impaired Antioxidative Protection

**DOI:** 10.1155/2016/5613056

**Published:** 2016-05-31

**Authors:** Matilde Otero-Losada, Hernán Gómez Llambí, Graciela Ottaviano, Gabriel Cao, Angélica Müller, Francisco Azzato, Giuseppe Ambrosio, José Milei

**Affiliations:** ^1^Institute of Cardiological Research, University of Buenos Aires, National Research Council (ININCA, UBA, CONICET), 1122 Buenos Aires, Argentina; ^2^University of Perugia School of Medicine, 06132 Perugia, Italy

## Abstract

We report experimental evidence confirming renal histopathology, proinflammatory mediators, and oxidative metabolism induced by cola drinking. Male Wistar rats drank* ad libitum* regular cola (C, *n* = 12) or tap water (W, *n* = 12).* Measures.* Body weight, nutritional data, plasma glucose, cholesterol fractions, TG, urea, creatinine, coenzyme Q_10_, SBP, and echocardiograms (0 mo and 6 mo). At 6 months euthanasia was performed. Kidneys were processed for histopathology and immunohistochemistry (semiquantitative). Compared with W, C rats showed (I) overweight (+8%, *p* < 0.05), hyperglycemia (+11%, *p* < 0.05), hypertriglyceridemia (2-fold, *p* < 0.001), higher AIP (2-fold, *p* < 0.01), and lower Q_10_ level (−55%, *p* < 0.05); (II) increased LV diastolic diameter (+9%, *p* < 0.05) and volume (systolic +24%, *p* < 0.05), posterior wall thinning (−8%, *p* < 0.05), and larger cardiac output (+24%, *p* < 0.05); (III) glomerulosclerosis (+21%, *p* < 0.05), histopathology (+13%, *p* < 0.05), higher tubular expression of IL-6 (7-fold, *p* < 0.001), and TNF*α* (4-fold, *p* < 0.001). (IV) Correlations were found for LV dimensions with IL-6 (74%, *p* < 0.001) and TNF*α* (52%, *p* < 0.001) and fully abolished after TG and Q_10_ control. Chronic cola drinking induced cardiac remodeling associated with increase in proinflammatory cytokines and renal damage. Hypertriglyceridemia and oxidative stress were key factors. Hypertriglyceridemic lipotoxicity in the context of defective antioxidant/anti-inflammatory protection due to low Q_10_ level might play a key role in cardiorenal disorder induced by chronic cola drinking in rats.

## 1. Introduction

Metabolic syndrome (MetS) is the constellation of hypertriglyceridemia, hyperglycemia, and/or insulin resistance, hypertension, and visceral obesity in man. In addition to increasing the risk for cardiovascular disease, diabetes, and diabetic nephropathy, MetS may directly affect renal morphology and/or function.

We have reported that chronic cola drinking induces MetS, pro-oxidative metabolism, and insulin resistance in rats and accelerates aortic atherosclerosis progression in adult ApoE^−/−^ mice as well [1–3].

The complex heart-kidney bidirectional dialogue involves mediators which, via bloodstream in the midst of the prevailing metabolic condition, reach target tissues and deliver specific messages. We also observed that MetS induced by chronic cola drinking might also involve renal pathology in normal rats (unpublished observations). Severity of MetS, posing a major risk factor for cardiovascular disease and type II diabetes, varies depending on the number of components of the syndrome itself. Yet, the connection of MetS with risk for renal impairment is not clear. Patients with MetS are at high risk for chronic kidney disease [4]. Cardiorenal syndrome can be generally defined as a pathophysiologic disorder of the heart and kidneys whereby acute or chronic dysfunction in one organ may induce acute or chronic dysfunction in the other organ [5]. By now, this condition is associated with significant morbidity and mortality, meeting the attention of both cardiologists and nephrologists.

Considering that cola drinking leads to metabolic changes which might individually affect heart and kidneys (e.g., severe hypertriglyceridemia and insulin resistance), the aim of this work was to evaluate whether chronic cola drinking may compromise kidney integrity in relation to oxidative metabolism and renal inflammation in rats.

## 2. Methods

Animal handling, maintenance, and euthanasia procedures were performed according to international recommendations [6]. The study was approved by the Committee of Ethics in Animal Research of the Instituto de Investigaciones Cardiológicas and the Institutional Animal Care and Use Committee (CICUAL) of the Faculty of Medicine of the University of Buenos Aires. Animals were housed at the institute facilities (21 + 2°C, at 12 h light-dark cycles, 7 a.m.–7 p.m.) and were fed a commercial chow (16%–18% protein and 0.2 g% sodium (Cooperación, Buenos Aires, Argentina))* ad libitum*.

### 2.1. Experimental Protocol

Twenty-four male Wistar rats were randomly distributed in 2 groups, according to beverage offered as the only liquid source (*ad libitum*): W (water) or regular cola (C) (commercially available sucrose-sweetened carbonated drink, Coca-Cola*™*, Argentina). Food and drink consumption were assessed twice a week, body weight was determined weekly, and systolic blood pressure (SBP) was recorded biweekly. At baseline and 6 months after the beginning of the study, biochemical analyses were performed and echocardiograms (awake rats) were recorded. After 6 months of drinking treatment, all the animals were euthanized by subtotal exsanguination under anesthesia (sodium thiopental 40 mg/kg, i.p.) and kidneys were excised for histopathology and immunohistochemistry.

According to company specifications Coca-Cola is a carbonated water solution containing (approximate%) 10.6 g carbohydrates, sodium 7 mg, caffeine 11.5 mg, caramel, phosphoric acid, citric acid, vanilla extract, natural flavorings (orange, lemon, nutmeg, cinnamon, coriander, etc.), lime juice, and fluid extract of coca (*Erythroxylum novogranatense*). Cola drink had carbon dioxide content largely removed by vigorous stirring using a stirring plate and placing a magnetic bar in a container filled with the liquid prior to being offered to the animals at room temperature.

### 2.2. Biochemical Determinations

Plasma levels of glucose, cholesterol fractions, triglycerides (TG), urea, and creatinine were determined in blood samples collected from the tail vein after 4-hour fasting, using commercially available kits for enzymocolorimetry (Sigma-Aldrich, USA) [7]. Atherogenic index of plasma (AIP) was calculated as AIP = log (TG/HDL − total Ch).

Plasma concentration of the lipophilic antioxidant ubiquinone-10 (2,3 dimethoxy-5 methyl-6-decaprenyl benzoquinone-10, coenzyme Q_10_) was measured using reversed phase-high performance liquid chromatography with ultraviolet detection (RP-HPLC-UV) at absorbance wavelength 275 nm [8].

### 2.3. Blood Pressure Determination

Systolic blood pressure (SBP) was measured by tail cuff plethysmography in awake rats gently restrained in a plastic chamber. The average of at least 3 readings per session was recorded. A pneumatic pulse transducer positioned on the ventral surface of the tail, distal to the occlusion cuff, detected the return of the pulse wave following a slow deflation of the cuff. Cuff pressure was determined by a pneumatic pulse wave transducer, using a programmed electrosphygmomanometer PE-300 connected to a Physiograph MK-IIIS for pulse recording (Narco Bio-Systems, Austin, Texas).

### 2.4. Echocardiography

Transthoracic echocardiograms were obtained in awake, gently restrained rats using an ATL 3000 HDI (Bethold, WA, USA) echocardiographic system equipped with a 10.5 MHz transducer. Echocardiography images (M-mode and 2-dimensional) were acquired in short axis views at the level of papillary muscle. Interventricular septal end diastolic dimension (IVSd) and left ventricular end diastolic posterior wall dimension (LVPWd) were determined at the parasternal long axis at midchordal level. Left ventricular diastolic dimension (LVDD) and left ventricular end systolic posterior wall dimension (LVPWs) were measured perpendicularly to the long ventricular axis also at midchordal level.

Typical echocardiographical parameters were calculated: shortening fraction (Sf%) = 100 × (LVPWd − LVPWs)/LVDD; left ventricular mass (LVM) = (LVDD + RWTh + LVPWd)^3^  − (LVDD)^3^  × 1.04; relative posterior wall thickness (RWTh) = (LVPWd + RWTh)/LVDD; end diastolic volume (EDV) = 0.85 × (LVDD)^3^; end systolic volume (ESV) = 0.85 × (LVSD)^3^; cardiac output = (EDV − ESV)^*∗*^  × heart rate; systolic volume (SV) = EDV − ESV. Echocardiographic images and heart rate (HR) were simultaneously recorded.

### 2.5. Histopathology and Quantitative Morphology

Kidneys were immediately dissected out after euthanasia, perfused with saline through the renal vein, weighed, and longitudinally cut. After fixation in phosphate buffered 10% formaldehyde (pH = 7.2) for 24 h, tissue pieces were embedded in paraffin, cut out into 4 *μ*m thick sections, and routinely stained with hematoxylin-eosin (HE) and periodic acid-Schiff (PAS). Tissue sections were examined under a light microscope (Nikon Eclipse 50i, Nikon Corporation, Tokyo, Japan) for the presence of histopathological changes. Images were captured, converted to digital photomicrographs (Nikon Coolpix S4), and analyzed using the Image-Pro Plus image processing software 6.0 (Media Cybernetics, Silver Spring, Maryland, USA). Histopathological evaluation was blinded to the experimental group.

Kidney sections were classified according to the presence and severity of glomerular, tubular, vascular, and interstitial abnormalities using a semiquantitative scale from 0 (zero) indicating no alterations through 1+, 2+, 3+, and 4+ indicating mild, moderate, moderately severe, and severe abnormalities, respectively. An overall histological score for each kidney was obtained [9].

Glomerular volume (Vg, 10^6^ *μ*m^3^) was estimated based on maximal planar area (MPA) analysis which was performed using the point-counting method. An orthogonal grid with 300 test points, representing an area of 6.7 10^4^ *μ*m^2^ at 40x objective lens, projected onto the fields of view. The number of points hitting the glomeruli (*n*) was counted in ≥50 glomeruli/kidney and used to calculate MPA (*μ*m^2^) as = *n* × *d* ^2^, where *d* is between-points distance [10].

Glomerular lesions were defined by the presence of focal and segmental glomerular scarring and obliteration of glomerular capillaries with increased mesangial cellularity, mesangial matrix expansion, and adhesion formation between the tuft and Bowman's capsule. Severity of glomerulosclerosis was semiquantitatively determined by Raij's method [11].

Image analysis was performed using a Nikon Eclipse 50i microscope (Nikon Corporation, Tokyo, Japan), incorporating a digital camera (Nikon Coolpix S4) and the Image-Pro Plus image processing software 6.0 (Media Cybernetics, Silver Spring, Maryland, USA).

### 2.6. Immunohistochemistry

The traditional avidin-biotin-peroxidase complex technique was used and a semiquantitative score allowed determination of immunohistochemical labelling of specimens [12]. Tubular staining for thioredoxin-1 (Trx1) (TTrx1), peroxiredoxin-2 (Prx2) (T_Prx2_), interleukin (IL)-6 (T_IL-6_), and tumor necrosis factor-alpha (T_TNF-*α*_) was performed using respective primary polyclonal rabbit antibodies. Control sections were incubated with nonimmune normal rabbit serum. Intensity of immunochemistry positivity was determined by the integrated optical density (IOD) method using the Image-Pro Plus image processing software 6.0 (Media Cybernetics, Silver Spring, Maryland, USA).

### 2.7. Statistical Analysis

Gaussian distribution was assessed by the Kolmogorov and Smirnov method. For variables with a Gaussian distribution (parametric), values were analyzed by two-way ANOVA followed by* post hoc* tests (Bonferroni multiple *t*-test) in order to evaluate between-groups' differences. Pearson correlation test was used to evaluate associations between variables (SPSS*™* 15.0). For variables with non-Gaussian distribution (histological scores), values were analyzed using the Kruskal-Wallis test (non-parametric analysis of variance) and Dunn's multiple comparison test for between-group comparisons. A value of *p* < 0.05 was considered significant in all cases (GraphPad Prism 5.0, GraphPad Software, Inc., San Diego, California, USA).

## 3. Results

**Figure 1:**
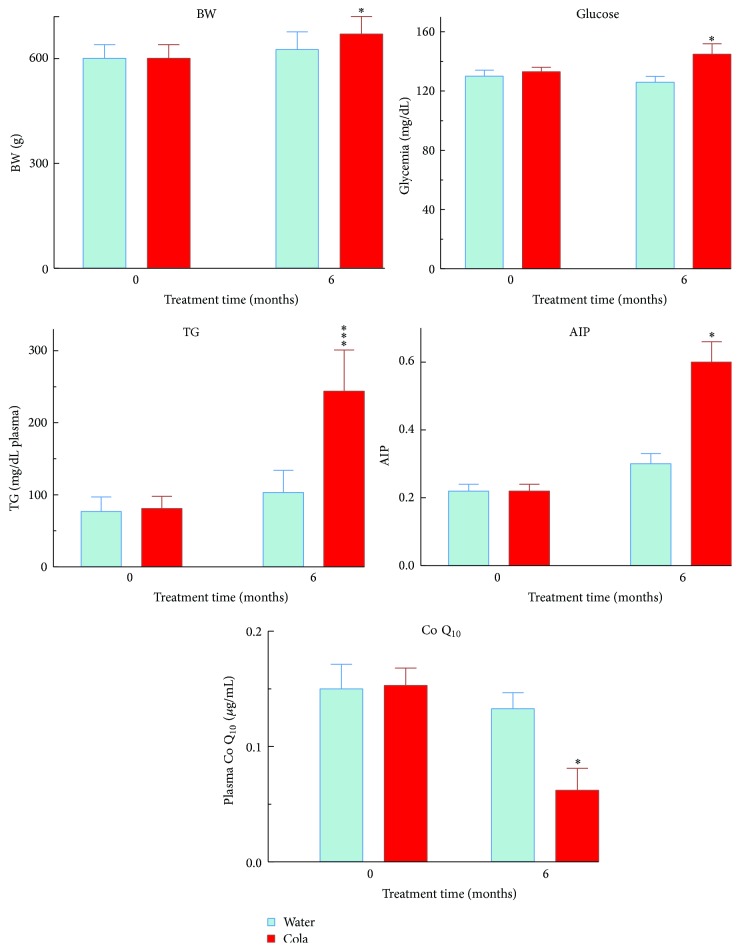
Body weight, biochemical profile (general metabolism), and Q_10_ level before and after cola treatment. BW: body weight, TG: triglycerides, AIP: atherogenic index in plasma, and Q_10_: coenzyme Q_10_ (ubiquinone Q_10_). ^*∗*^ *p* < 0.05 and ^*∗∗∗*^ *p* < 0.001. Compared with W, C rats showed overweight (+8%, *p* < 0.05), hyperglycemia (+11%, *p* < 0.05), hypertriglyceridemia (2-fold, *p* < 0.001), higher AIP (2-fold, *p* < 0.01), and lower Q_10_ levels (−55%, *p* < 0.05).

**Figure 2:**
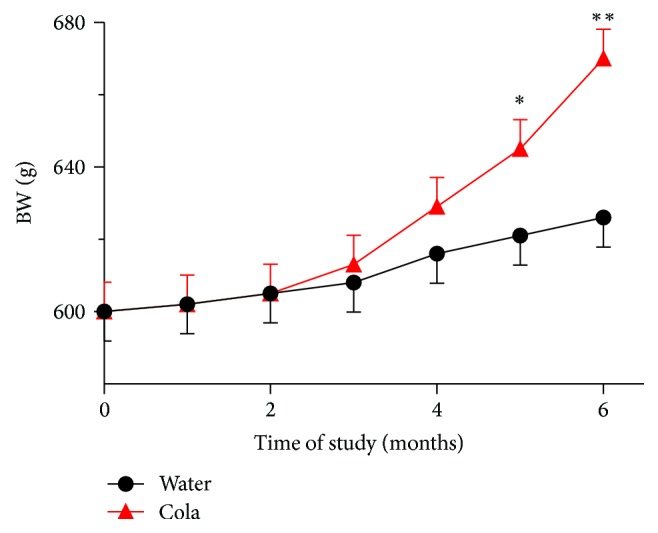
Body weight (BW) over the time of study. ^*∗*^ *p* < 0.05 and ^*∗∗*^ *p* < 0.01 versus water drinking group.

After 6 months of cola drinking (C), rats showed large drinking volumes (mL/kg/24 hs) (150 ± 28 in C versus 87 ± 12 in W, *p* < 0.001) and developed overweight (+8%, *p* < 0.05), hyperglycemia (+11%, *p* < 0.05), hypertriglyceridemia (2-fold, *p* < 0.001), higher AIP (2-fold, *p* < 0.01), and lower Q_10_ levels (−55%, *p* < 0.05) compared with their water drinking counterparts (W) (Figure 1). Between-group difference in body weight increase over time became statistically significant only beyond 5 months of treatment (*p* < 0.05 at 5 months; *p* < 0.01 at 6 months, Figure 2). The decrease in Q_10_ concentration was 81% accounted for by the increase in TG and vice versa (*r* = 0.90, *p* < 0.01). Consumption of cola drinks did not modify either uremia (mg/100 mL) 33.3 ± 3 in C versus 26 ± 4 in W or creatinine (mg/100 mL: 0.53 ± 0.02 in C versus 0.58 ± 0.04, N.S.).

**Figure 3:**
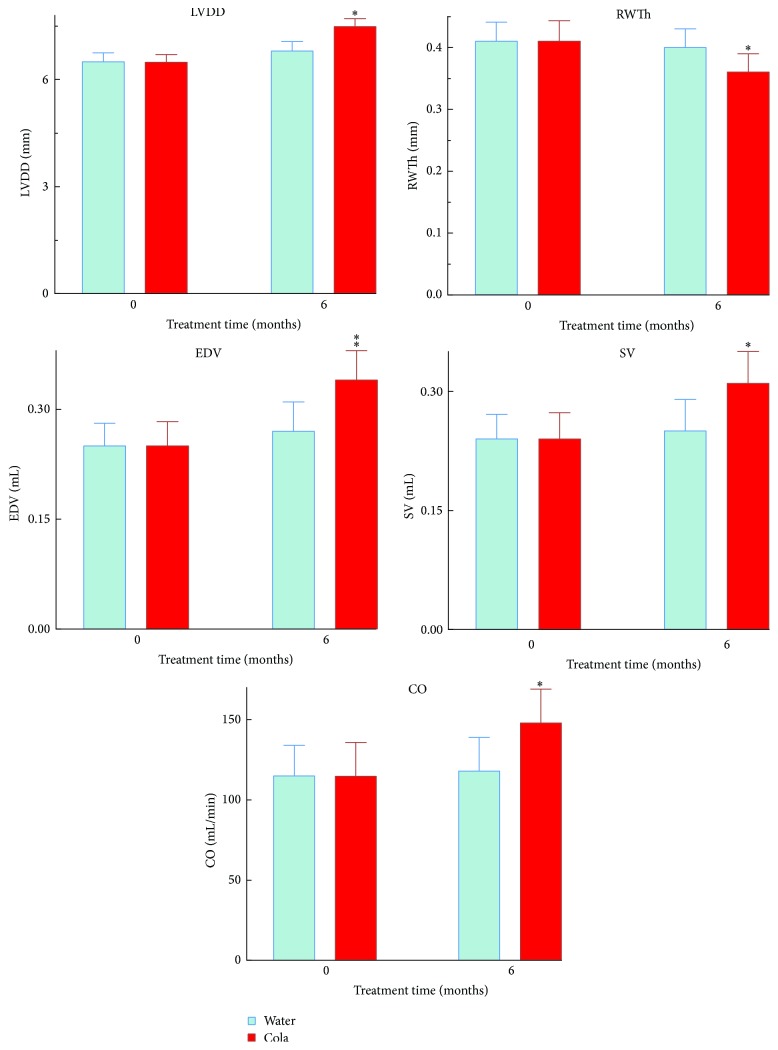
Echocardiographic parameters in rats before and after cola treatment. LVDD: left ventricle diastolic diameter, SV: stroke volume, RWTh: relative posterior wall thickness of LV, EDV: end diastolic volume, CO: cardiac output, and SBP: systolic blood pressure. ^*∗*^ *p* < 0.05 and ^*∗∗*^ *p* < 0.01 compared with water.

Echocardiographical analysis revealed that compared with W rats, C rats showed increased LV diastolic diameter (+9%, *p* < 0.05) and increased both LV diastolic volume (+26%, *p* < 0.01) and LV systolic volume (+24%, *p* < 0.05). Posterior wall thinning (−8%, *p* < 0.05) with larger cardiac output (+24%, *p* < 0.05) and no change in heart rate (HR) were also found in C rats compared with W rats (Figure 3).

**Figure 4:**
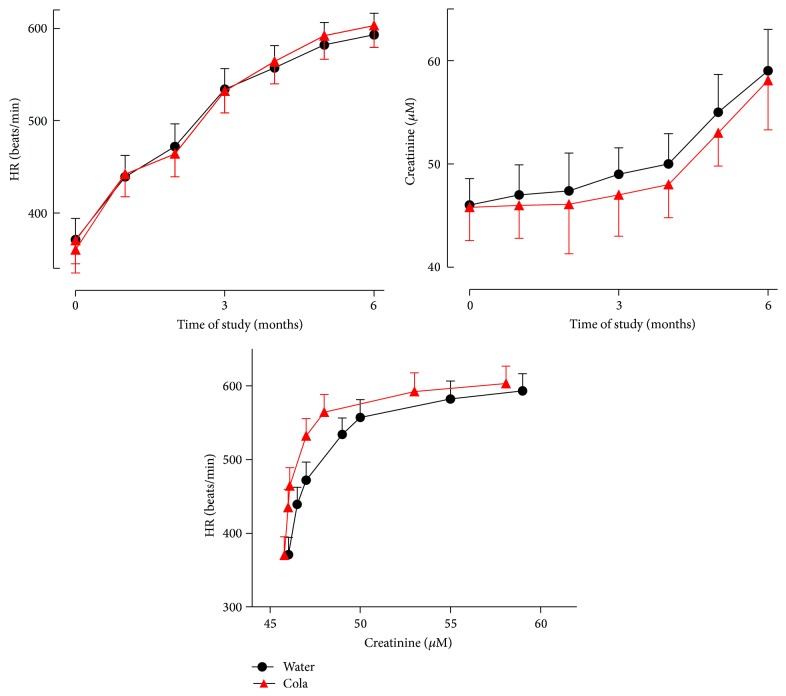
Heart rate (HR), creatinine, and their relationship over the time of study. Cola drinking did not affect HR, creatinine, or HR-creatinine association over time.

Cola consumption had no effect on either HR or creatinine and did not disrupt the relationship between HR and creatinine over time (Figure 4).

**Figure 5:**
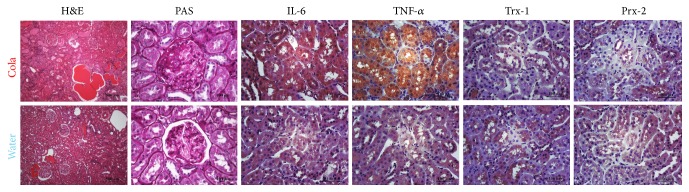
Immunohistochemistry of renal glomeruli and tubules before and after cola treatment. Cola treatment (C, top row) resulted in glomerulosclerosis (PAS column) and intense tubular immunopositivity for IL-6 and TNF-*α*, with no major change in redoxins Trx-1 and Prx-2 immunopositive labelling compared with water drinking rats (W, bottom row). H&E: hematoxylin-eosin, PAS: periodic acid-Schiff, Trx-1: thioredoxin-1, Prx-2: peroxiredoxin-2, IL-6: interleukin-6, and TNF-*α*: tumor necrosis factor-alpha.

Microphotographs of renal tissue revealed focal segmental glomerulosclerosis and intense tubular immunopositivity for IL-6 and TNF-*α* after 6 months of sustained cola drinking (Figure 5).

**Figure 6:**
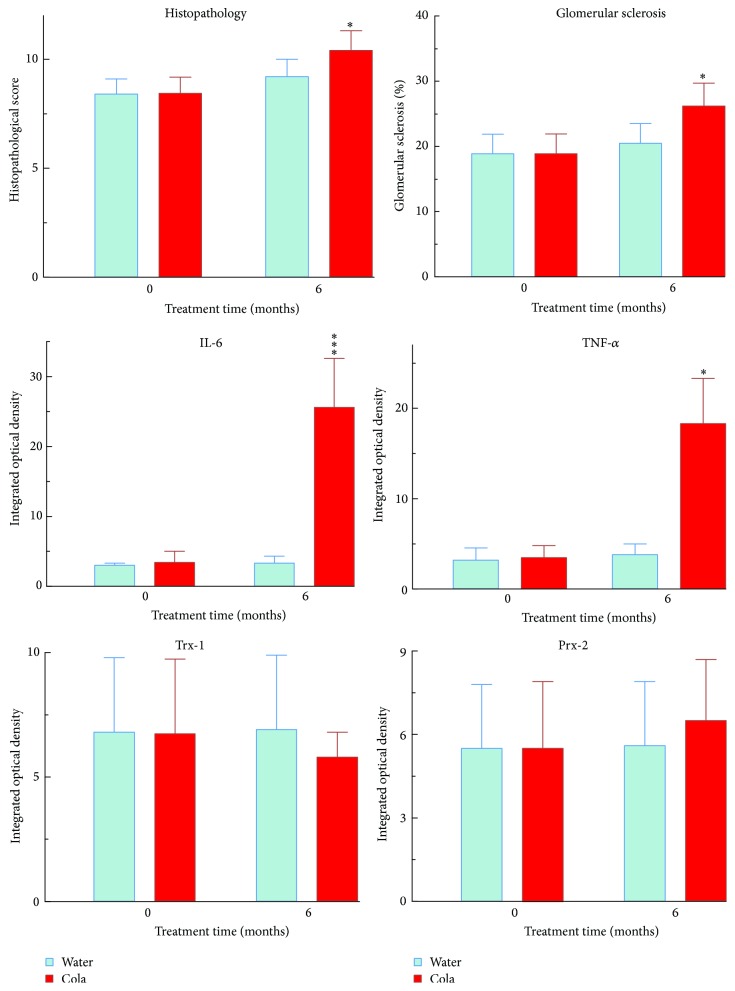
Renal glomerular pathology, inflammatory cytokines, and redoxins in renal tubules before and after cola treatment. IL-6: interleukin-6, TNF-*α*: tumor necrosis factor-alpha, Trx-1: thioredoxin-1, and Prx-2: peroxiredoxin-2. ^*∗*^ *p* < 0.05 and ^*∗∗∗*^ *p* < 0.001 compared with water.

Cola drinking treatment induced glomerulosclerosis (+21%, *p* < 0.05), higher histopathological score (+13%, *p* < 0.05), and largely higher tubular expression of both IL-6 (7-fold, *p* < 0.001) and TNF-*α* (4-fold, *p* < 0.001) (Figure 6).

**Figure 7:**
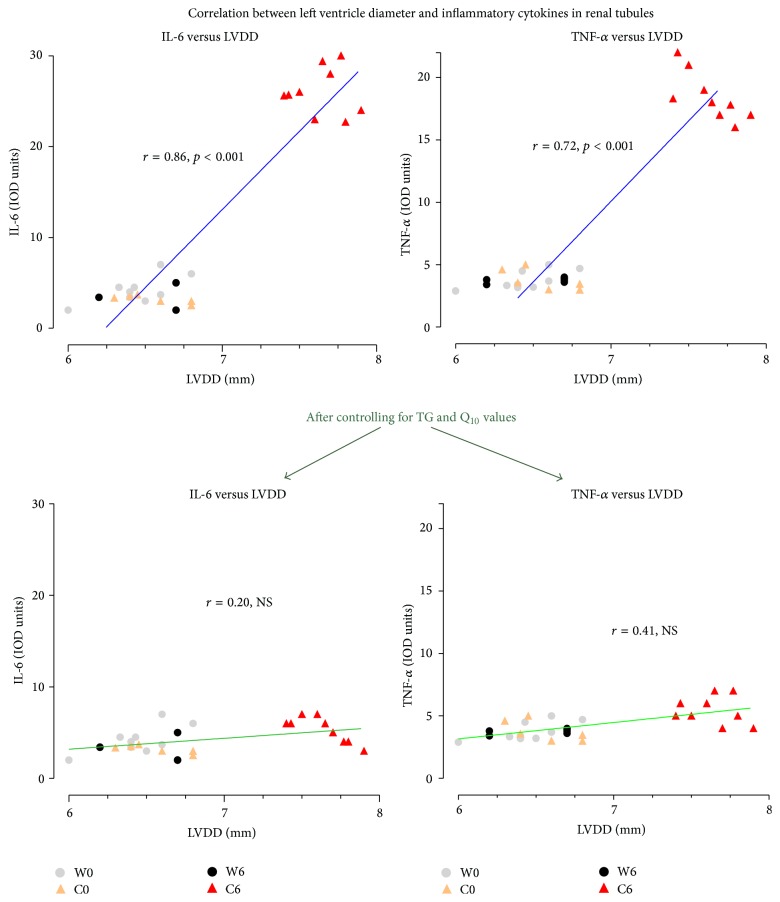
Cardiorenal correlations. Dependence on TG and Q_10_ levels. IL-6: interleukin-6, and TNF-*α*: tumor necrosis factor-alpha.

Correlations were found for changes in LV dimensions with IL-6 (74%, *r* = 0.86, and *p* < 0.001) and TNF-*α* (52%, *r* = 0.72, and *p* < 0.001). Controlling for either TG or Q_10_ values individually reduced the strength of correlations to (% of mutually explained variance) 22%, *r* = 0.47, and *p* < 0.05 for IL-6 and 14%, *r* = 0.38, and *p* < 0.05 for TNF-*α*. Moreover, controlling for both TG and Q_10_ levels altogether actually abolished any correlation previously observed for LV dimensions with IL-6 (*r* = 0.20, NS) and TNF-*α* (*r* = 0.41, NS) (Figure 7).

## 4. Discussion

In the present paper, the striking increase in triglycerides following regular cola consumption can be explained by high content of fructose and large drinking volumes in C group. Interestingly, the decrease in Q_10_ was 81% accounted for by the increase in TG and vice versa revealing an intimate and bidirectional metabolic connection. Hypertriglyceridemia, increasing the demand of antioxidant factors to protect against further lipoperoxidation, might be responsible for exhaustion of the mitochondrial production of Q_10_ level. Q_10_ level has been suggested to be a useful biomarker of oxidative stress [13]. In this regard, MetS is associated with higher levels of circulating oxidized LDL [14]. Cola drinking induced left ventricle hypertrophy (LVH), namely, larger diastolic and systolic volumes with posterior wall thinning, increased stroke volume, and cardiac output without affecting heart rate, likely as a result of LVH and a rise in preload (EDV) and afterload [15]. On one hand, these changes may be partly explained by fluid overload after drinking large volumes in C group. Ingestion of large volumes of cola, a carbohydrate-rich hypertonic solution having 493 mOsm/L compared with 285–295 mOsm/L of plasma or 3 mOsm/L in hypotonic tap water, is expected to increase blood volume and CO, through sequestration of fluid from intracellular compartments.

Cola drinking may stimulate hypothalamic antidiuretic hormone (ADH, vasopressin) secretion and increase blood volume in order to keep physiological osmolarity in plasma. The driving force responsible for the movement of fluid into the interstitial space is regulated by circulating factors, mainly glucose drawn from the splanchnic circulation [16]. Interestingly, stimulation of ADH release would stimulate thirst in C rats, helping to explain the large drinking volumes observed in this group in addition to the sweet taste preference behaviour.

On the other hand, low Q_10_ levels in plasma have been associated with cardiac hypertrophy [17]. Oppositely, Q_10_ has been reported to have a direct antihypertrophic effect on rat cardiomyocytes in vitro and combining Q_10_ with low-dose losartan provided additive therapeutic benefit, reducing hypertension and LVH [18].

Actually MetS is clearly linked to Q_10_ deficiency [19]. In experimental and human diabetic nephropathy, advanced glycation end products (AGEs) accumulate with malondialdehyde in glomerular lesions in relation to disease severity and in the presence of an upregulated receptor for AGE (RAGE) in podocytes [19]. Toxic effects of AGEs result from structural and functional alterations via cross-linking of plasma and extracellular matrix proteins. In mesangial and endothelial cells, AGE-RAGE interaction causes enhanced formation of oxygen radicals with subsequent activation of nuclear factor-kappaB and release of the proinflammatory cytokines IL-6 and TNF-*α* [19].

Insofar, the effects of cola intake on glomerular structure might be secondary to metabolic syndrome and/or they might be related to other factors as well, such as increased fluid overload and intravascular expansion as noted in our previous report [1]. Present results might appear as in apparent discrepancy with our earlier observations showing kidney lesions attributable to the aging process in cola drinking rats. However, thorough histopathology examination was not performed in that study.

The more we advance in the study of the effects of chronic cola drinking, the more we meet new pieces of the multifactorial puzzle upstream metabolic syndrome manifestations and long-term complications. Actually, metabolic syndrome actually poses a threat to kidney structure and function in the long run [20].

The increase in creatinine as a function of time (age of the animals) is interpreted in terms of the functional status of the kidney and its deterioration over time. Cola consumption did not affect creatinine and most important did not affect heart rate-creatinine relationship (the shape of the association curve was unaltered) suggesting that the kidney responded adequately to variation in heart rate over 6 months of cola drinking. The relationship between creatinine and heart rate has been reported [21].

On the other hand, since rats develop insulin resistance over 6 months of cola drinking as we reported [3] and present results show an increase of inflammatory mediators in renal tubules, the possibility that 6 months of cola drinking might predispose to mild renal insufficiency in due time cannot be ruled out until experimental confirmation. Mild renal insufficiency is associated with inflammation and insulin resistance [22]. Epidemiological studies have shown an association between the intake of cola beverages and chronic kidney disease [23].

In a previous study, 3-month cola drinking did not affect body weight, glomerular morphology, or oxidative status in renal cortex [23]. In contrast, in our study, mild overweight and glomerular histopathology were observed after 6-month cola drinking with no change in the immunohistochemical expression of thioredoxins, in agreement with the previous study. Cola consumption in that study (average 140 mL/day) was similar to cola intake in the present study and both studies evaluated male Wistar rats. However treatment length was largely different and, in this experimental model as in many others, changes over time are juxtaposed to changes due to time (age) itself. For instance, present difference in body weight in cola drinking rats achieved statistical significance at 5 months, not before. Hence differences between the two studies are interpreted mainly in terms of time-dependence and treatment length.

The dramatic increase in proinflammatory cytokines IL-6 and TNF-*α* in renal tubules induced by cola drinking rats should result in tubular derangement and dysfunction if sustained in time (not the aim of this study). Experimental and clinical studies have suggested a correlation between the progression of renal disease and dyslipidemia. Hypertriglyceridemia elicits inflammatory responses in different tissues and is known to affect morphological integrity of kidney [24]. Dyslipidemia and lipotoxicity-induced insulin resistance, inflammation, and oxidative stress are the key pathogeneses of renal damage in type 2 diabetes [25]. Interestingly, we have reported development of insulin resistance in cola drinking rats [3]. In reasonable agreement with present evidence and previous reports, we suggest that MetS induced by cola drinking affects kidney structure and increases proinflammatory cytokines in renal tubules in rats and that hypertriglyceridemia and low levels of Q_10_ may play crucial roles in determining the pathophysiology of the cardiorenal axis. High content of advanced glycation end products (AGEs) in caramel colorant in cola beverages further perpetuates oxidative stress, contributing to the increase in proinflammatory cytokines in renal tubules and may be involved in the progression to chronic kidney disease as one of the complications of MetS in cola beverages consumers [26].

Five subtypes of cardiorenal alterations have been identified according to pathophysiology, time-frame, and the nature of concomitant renal dysfunction. Cola drinking might be considered to induce a metabolic condition that goes beyond the typical MetS and may progress to a type 5 cardiorenal alteration in due time (i.e., renal and cardiac dysfunction due to a systemic metabolic condition) [27].

Chronic kidney disease is an emerging health problem but only few patients would reach end renal stage. There exists an increasing strong association between MetS and chronic kidney disease though the connection between them is unclear and there are few studies showing renal histology in MetS [28]. Acute kidney injury has been recently reported in a patient with metabolic syndrome with previous normal kidney function [28]. In this paper, we present evidence showing that MetS induced by cola drinking affects renal structure in rats and increases the level of proinflammatory cytokines IL-6 and TNF-*α* in renal tubules, in the context of severe hypertriglyceridemia and a decrease in the antioxidant/anti-inflammatory Q_10_ levels.

## 5. Conclusion

Chronic cola drinking induced cardiac remodeling associated with increase in proinflammatory cytokines and renal damage. Cardiorenal association was dependent on hypertriglyceridemia and oxidative stress. Hypertriglyceridemic lipotoxicity in a context of defective antioxidant and anti-inflammatory protection due to low Q_10_ level might be involved in the cardiorenal syndrome induced by chronic cola drinking in rats.

Based on present findings and according to the classification by Ronco et al. [5], experimental MetS induced by chronic cola drinking, presenting cardiac hypertrophy and renal histopathology (glomerular sclerosis) [1, 2], may provide an interesting model to study type 5 cardiorenal syndrome as well.
